# Differential effects of curcumin on vasoactive factors in the diabetic rat heart

**DOI:** 10.1186/1743-7075-3-27

**Published:** 2006-07-18

**Authors:** Hana Farhangkhoee, Zia A Khan, Shali Chen, Subrata Chakrabarti

**Affiliations:** 1 Department of Pathology, The University of Western Ontario, London, Ontario N6A 5C1, Canada; 2 Vascular Biology Program and Department of Surgery, Children's Hospital Boston, Harvard Medical School, MA 02115, USA

## Abstract

**Background:**

Increased oxidative stress has been associated with the pathogenesis of chronic diabetic complications, including cardiomyopathy. Recent studies indicate that curcumin, a potent antioxidant, may be beneficial in preventing diabetes-induced oxidative stress and subsequent secondary complications. We have investigated the effects of curcumin on the nitric oxide (NO) pathway in cardiac tissues and cultured cells.

**Methods:**

Streptozotocin-induced diabetic rats were treated with curcumin for a period of one month. Heart tissues were then analyzed for endothelial NO synthase (eNOS) and inducible NO synthase (iNOS) mRNA expression. Oxidative protein and DNA damage were assessed by immunohistochemical analysis of nitrotyrosine and 8-hydroxy-2'-deoxyguanosine (8-OHdG). Heart tissues were further subjected to endothelin-1 (ET-1) mRNA expression. In order to further characterize the effects of curcumin, we assayed microvascular endothelial cells (MVECs). Cultured MVECs, exposed either to glucose or glucose and varying concentrations of curcumin, were assessed for alterations of NOS expression and activation of nuclear factor-κB (NF-κB) and activating protein-1 (AP-1). Oxidative stress and ET-1 expression levels were also assayed.

**Results:**

Our results indicate that one month of diabetes causes an upregulation of both eNOS and iNOS mRNA levels, and nitrotyrosine and 8-OHdG immunoreactivity in the heart. Treatment of diabetic rats with curcumin reduced eNOS and iNOS levels in association with reduced oxidative DNA and protein damage. Interestingly, curcumin further increased vasoconstrictor ET-1 in the heart. Exposure of MVECs to high glucose increased both eNOS and iNOS levels and oxidative stress. Curcumin prevented NOS alteration and oxidative stress in a dose-dependent manner which was mediated by nuclear factor-κB and activating protein-1. Exposure to curcumin also increased ET-1 levels in the MVECs.

**Conclusion:**

Our studies indicate the differential effects of curcumin in vasoactive factor expression in the heart and indicate the importance of tissue microenvironment in the treatment of diabetic complications.

## Background

Nearly 40% of diabetic patients develop secondary complications [1-3]. These complications arise primarily due to sustained hyperglycemia [4,5]. Among the vast array of problems associated with long-standing diabetes, cardiovascular complications, including diabetic cardiomyopathy, have been clearly documented as detrimental and as one of the leading causes of mortality [1-3]. Over the years, research has identified some pathological mechanisms underlying diabetic complications [6-9]. One of the purposed mechanisms is increased oxidative stress via augmentation of reactive oxygen species (ROS) [6-9].

An interesting pathway suggested to be involved in augmenting oxidative stress is the nitric oxide (NO) pathway [10,11]. NO is produced by a set of three nitric oxide synthase (NOS) isozymes: endothelial NOS (eNOS), inducible NOS (iNOS), and neuronal NOS (nNOS) [12,13]. These enzymes convert L-arginine to L-citrulline, leading to the generation of the free radical NO [12,13]. NO is very reactive and is readily sequestered by superoxide anions to form peroxynitrite. Peroxynitrite has been shown to damage proteins by modifying the tyrosine residues [14,15]. Many studies have shown that eNOS and iNOS are important players in the pathogenesis of diabetic cardiovascular complications [8,16-18]. Our previous studies indicate that diabetes leads to increased eNOS and iNOS levels in the heart, whereas the amount of NO remains unaltered [18]. It is plausible that NO is quickly scavenged by free radicals such as superoxide anions, producing peroxynitrite.

Curcumin, the active component in Tumeric Rhizomes (*Curcuma Long Linn*), was originally used in tradition Indian medicine over 3000 years ago [19]. Several studies have indicated a beneficial role of curcumin in terms of antioxidant, anti-tumourgenic, and anti-inflammatory property [20]. A recent study showed that curcumin-treated diabetic rats had lower blood glucose and glycated hemoglobin levels, in association with lower oxidative stress [21]. Furthermore, treatment with curcumin has been shown to reduce ROS levels in cells isolated from diabetic patients [22]. It has been suggested that curcumin may mediate some of the effects by partial inhibition of iNOS via nuclear factor-κB (NF-κB) [20,23,24]. Hence, we investigated the role of curcumin on diabetes-induced vasoactive factor alteration including NO to elucidate the possible mechanisms underlying the antioxidant activity. We have further examined the effect of curcumin in cultured endothelial cells.

## Methods

### Animal model of chronic diabetes

Male Sprague-Dawley rats (Charles River Canada Ltd., QC Canada), weighing approximately 200–250 g, were randomly divided into three groups (n = 6): controls (CO), diabetic animals (DM), and diabetic animals treated with curcumin (DM-C). Diabetes was induced by a single intravenous injection of streptozotocin (STZ; 65 mg/kg in citrate buffer, pH = 5.6) [25,26], while the control animals received the same volume of citrate buffer. The use of STZ to induce diabetes provides an animal model of type 1 diabetes, as the drug causes β-islet cell apoptosis. Curcumin was administered via intraperitoneal injection (150 mg/kg; Sigma-Aldrich, ON Canada) [27,28]. The rats were monitored daily for ketonuria and were given small doses of insulin (0.1 – 3 U) to prevent ketosis. After 4 weeks of treatment, animals were sacrificed and heart tissues were snap-frozen in liquid nitrogen for gene expression analysis and embedded in paraffin for immunohistochemical analysis. Clinical monitoring of the animals was also performed to evaluate body weight and blood glucose concentrations. All animals were cared for according to the Guiding Principle in the Care and Use of Animals. All experiments were approved by the University of Western Ontario Council on Animal Care Committee.

### Endothelial cells cultures

Human microvascular endothelial cells (HMECs; Clonetics, MD USA) were cultured at 2500 cells/cm^2 ^in endothelial growth medium (Clonetics) as previously described [29]. Cells were culture in 5 mM glucose (low glucose control; CO) and 25 mM glucose (high glucose; HG). In addition, cells cultured in HG were treated with varying concentrations of curcumin (0.1, 10, and 100 μM; HG-C) [30,31]. After 24 hours of treatment, RNA was extracted and subjected to Real-time RT-PCR. For subsequent analyses, cells were treated with empirically determined curcumin concentration.

### RNA extraction and Real Time RT-PCR

RNA was isolated from heart tissues and cultured ECs as described previously [18,32]. cDNA was synthesized with 3 μg of total RNA. The mRNA levels of eNOS, iNOS, and ET-1 were quantified using the LightCycler™ (Roche Diagnostics Canada, QC Canada) [18,32]. In each reaction tube the following reagents were added for a final volume of 20 uL: 10 μL of LC DNA Master SYBR Green 1 (Roche Diagnostic Canada), 1.6 μL of 25 mM of MgCl_2_, 1 μL each of 10 mM forward and reverse primers (Table 1), 5.4 μL of H_2_O, and 1μL of cDNA. PCR amplification protocols were optimized for ideal conditions as described previously [18,32]. For the assessment of early oxidative stress in cultured ECs [18,29,33], heme oxygenase-1 and -2 (HO-1, HO-2) were also determined.

**Table 1:** Primer sequences and PCR parameters

**Gene**	**Primers (5' → 3')**	Temperature*	
Rat eNOS	GCAAGACCGATTACACGACA GTCCTCAGGAGGTCTTGCAC	Denaturation Annealing Extension Signal	95°C-0 sec 57°C-5 sec 72°C-10 sec 85°C-1 sec
Human eNOS [42]	CCTCCAGGAAGGAGCAAAC TCCTGAGAGAGAGGCAAGAGGA	Denaturation Annealing Extension Signal	95°C-0 sec 55°C-5 sec 72°C-8 sec 81°C-1 sec
Rat iNOS	ATGGAACAGTATAAGCGAAACACC GTTTCCGGTCGATGTCATGAGCAAAGG	Denaturation Annealing Extension Signal	95°C-0sec 57°C-5 sec 72°C-10 sec 83°C-1 sec
Human iNOS [42]	CCCCATCAAGCCCTTTACTT CACCTCCTGGTGGTCACTT	Denaturation Annealing Extension Signal	95°C-0 sec 55°C-5 sec 72°C-8 sec 81°C-1 sec
Rat ET-1	GCTCCTGCTCCTCCTTGATG CTCGCTCTATGTAAGTCATGG	Denaturation Annealing Extension Signal	95°C-0 sec 55°C-5 sec 72°C-20 sec 84°C-1 sec
Human ET-1	AAGCCCTCCAGAGAGCGTTAT CCGAAGGTCTGTCACCAATGT 6FAM-TGACCCACAACCGAG-MGBNFQ	Denaturation Annealing Extension Signal	95°C-0 sec 55°C-5 sec 72°C-4 sec 72°C-1 sec
Human HO-1 [43]	TGATAGAAGAGGCCAAGA TTTCCAGACAGAGGGACA	Denaturation Annealing Extension Signal	95°C-0 sec 50°C-10 sec 72°C-17 sec 84°C-1 sec
Human HO-2 [43]	TGGAGCGCAACAAGGACCAT CCGGTAGAGCTGCTTGAACT	Denaturation Annealing Extension Signal	95°C-0 sec 50°C-10 sec 72°C-17 sec 84°C-1 sec
β-actin	CCTCTATGCCAACACAGTGC CATCGTACTCCTGCTTGCTG	Denaturation Annealing Extension Signal	95°C-0 sec 58°C-5 sec 72°C-8 sec 83°C-1 sec

* Initial denaturation was carried out at 95 °C for 3 minutes.

### Immunohistochemistry

Heart tissues were analyzed for nitrotyrosine, an oxidative protein damage marker, and 8-Hydrox-2'-deoxy Guanosine (8-OHdG), an oxidative DNA damage marker [18]. Paraffin-embedded heart tissues were sectioned (5 μm) and transferred to positively charged slides. Slides were stained using the Vectastain Elite Kit (Vector Laboratories, ON Canada) and monoclonal antibodies for nitrotyrosine (1:100, Caymen Chemicals, MI USA) and 8-OHdG (1:50, Japan Institute for the Control of Aging, Fukuroi, Japan). The chromagen, 3'-3' diaminobezine (DAB, Sigma-Aldrich Canada Ltd) was used to detect the oxidative protein and DNA damage levels. Staining with non-immune rabbit serum instead of primary antibodies was used as negative controls. Ten random fields were examined by two investigators unaware of the experimental treatment. Nitrotyrosine staining was evaluating by measuring the relative cytoplasmic staining intensity, while 8-OHdG was assessed by counting the number of positive cardiomyocytes.

### Electrophoretic mobility shift assay

Electrophoretic mobility shift assay (EMSA) was performed to assess activation of transcription factors, NF-κB and activating protein-1 (AP-1), as described previously [29,34].

### Statistical analysis

The data are expressed at mean ± S.E.M. and were analyzed by ANOVA followed by student's t-test. Statistical differences were considered when p < 0.05.

## Results

### Clinical monitoring of the diabetic animals

Animals were monitored for body weight gain and blood glucose levels to assess diabetic dysmetabolism. Diabetic animals exhibited decreased body weight gain and increased blood glucose levels as compared to non-diabetic controls (Table 2). Curcumin did not have any effect on these parameters.

**Table 2:** Clinical monitoring of animals

**Treatment**	**Body Weight Gain (gms)**	**Glucose (mM)**
CO	510 ± 20	4.2 ± 0.2
DM	435 ± 25*	22.1 ± 2.5*
DM-C	413 ± 2*	19.8 ± 1.4*

* p < 0.05 compared to CO

### Diabetes upregulates NOS and ET-1 levels in the heart with increased oxidative stress

**Figure 1:**
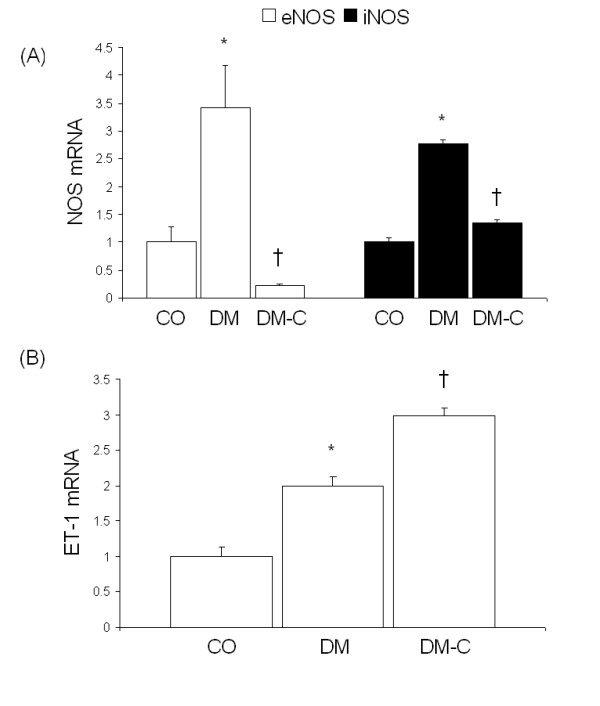
Effect of curcumin on diabetes-induced vasoactive factor expression, showing real time RT-PCR analysis of (A) NOS, and (B) ET-1 transcript levels in the heart tissues [CO, control; DM, diabetics; DM-C, diabetics treated with 150 mg/kg/d curcumin; *p < 0.05 compared to CO; †p < 0.05 compared to DM; n = 4/group].

**Figure 2:**
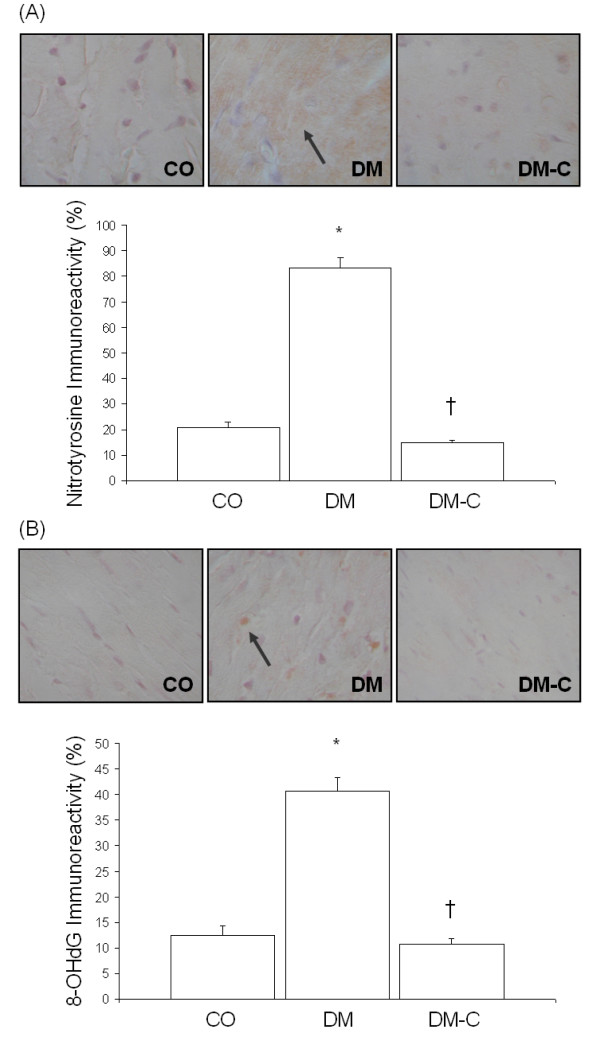
Diabetes-induced oxidative stress as assessed by (A) nitrotyrosine, and (B) 8-OHdG staining. Curcumin decreased the level of oxidative DNA and protein damage [Original magnification X400; *p < 0.05 compared to CO; †p < 0.05 compared to DM].

Heart tissues from diabetic animals exhibited increased eNOS and iNOS mRNA levels as compared to control rats (Figure 1A). ET-1 mRNA levels were also increased in the diabetic animals (Figure 1B). To determine whether alteration of NOS expression was associated with oxidative stress, we assayed for oxidative protein and DNA damage. Immunohistochemical analysis showed that diabetes caused increased nitrotyrosine immunoreactivity (Figure 2A). In addition, heart tissues from diabetic animals exhibited increased levels of 8-OHdG (Figure 2B).

### Curcumin prevents diabetes-induced NOS alteration and oxidative damage

Treatment of diabetic animals with curcumin prevented eNOS and iNOS mRNA upregulation (Figure 1A). Interestingly, curcumin caused a greater increase in diabetes-induced ET-1 expression in the heart (Figure 1B). We next determined whether the target organs of diabetic complications also exhibited increased ET-1 expression upon curcumin treatment. Analysis of retina and kidney samples revealed that ET-1 was normalized as compared to untreated diabetic animals (data not shown). The decrease in eNOS and iNOS levels was associated with reduced nitrotyrosine staining (Figure 2A), indicating decreased NO scavenging and oxidative protein damage. In addition, curcumin-treated diabetic animals showed decreased oxidative DNA damage (Figure 2B).

### Effect of curcumin on high glucose-induced eNOS and iNOS expression and oxidative stress in ECs

**Figure 3:**
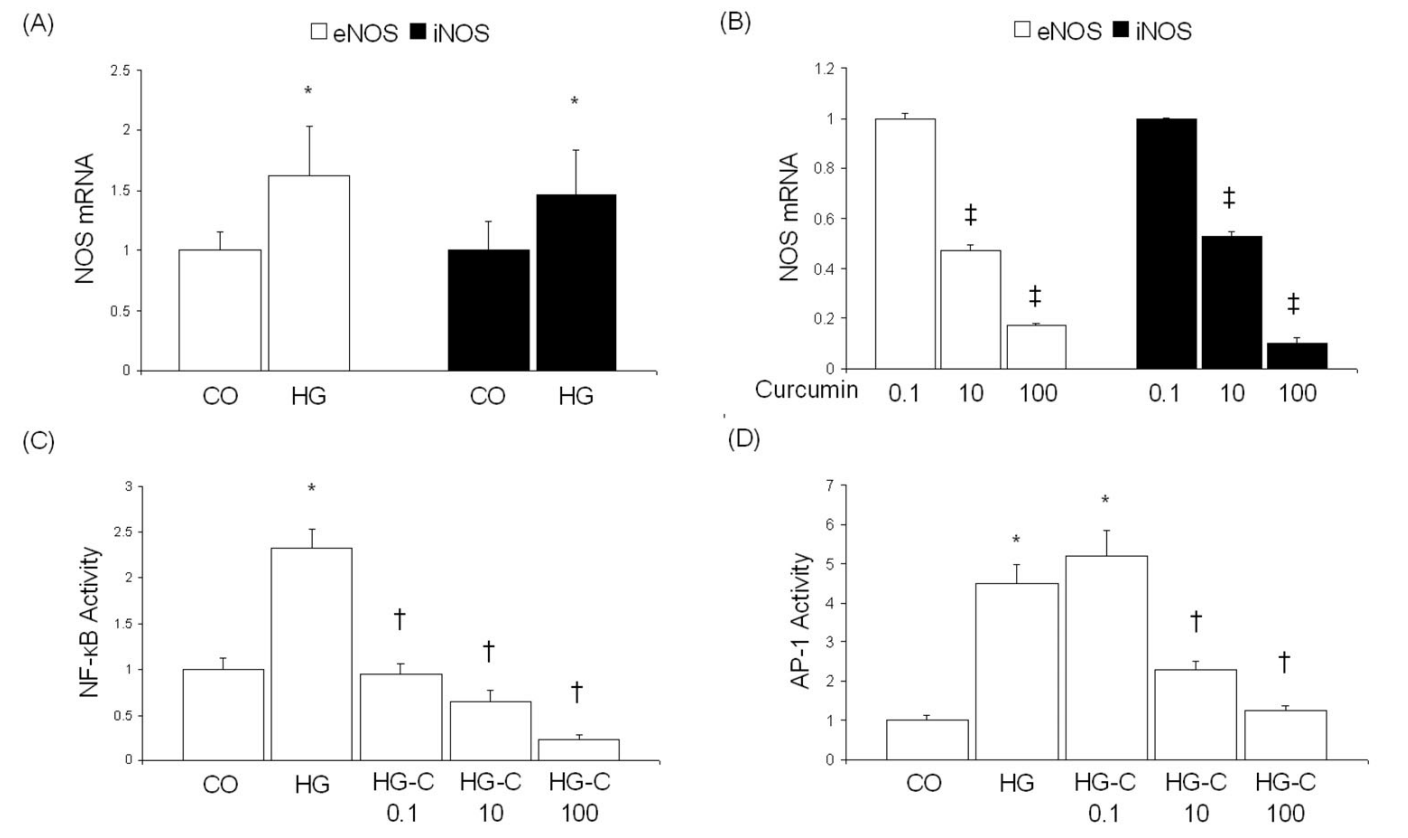
Effect of high glucose levels (A) and curcumin (B) on NOS mRNA levels and transcription factor activity in ECs. ECs exposed to 25 mM glucose were treated with varying curcumin concentrations (0.1, 10, and 100 μM in B) and assayed for eNOS and iNOS expression (A and B) and transcription factor activity (C and D) [CO, 5 mM glucose; HG, 25 mM glucose; HG-C, HG + curcumin (μM); *p < 0.05 compared to CO; †p < 0.05 compared to HG; ‡p < 0.05 compared to 0.1 μM].

**Figure 4:**
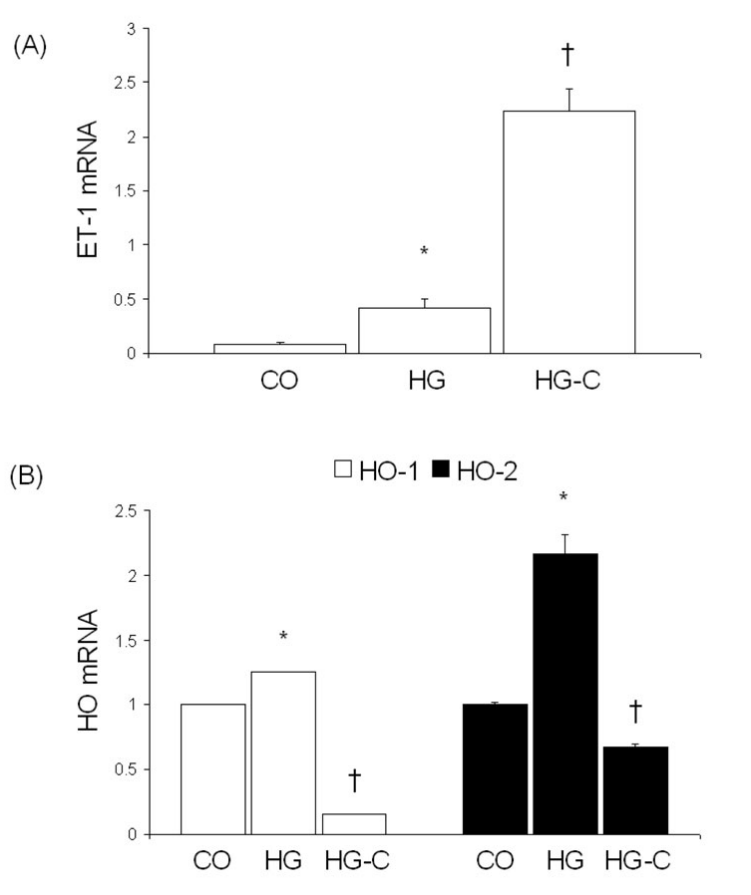
ET-1 (A) and HO (B) expression in ECs cultured with 25 mM glucose and 100 μM curcumin as assessed by real time RT-PCR [HG-C, HG + 100 μM curcumin; *p < 0.05 compared to CO; †p < 0.05 compared to HG].

In order to investigate the mechanisms of curcumin-mediated downregulation of NOS and oxidative stress, we assayed microvascular ECs. Exposure of ECs to high glucose levels (25 mmol/L) increased both eNOS and iNOS mRNA levels (Figure 3A). Treatment of cells exposed to 25 mM glucose to varying levels of curcumin prevented both eNOS and iNOS expression (Figure 3B). Such changes were mediated by decreased activation of redox-sensitive transcription factors, NF-κB and AP-1 (Figure 3C and 3D). To determine whether curcumin alters ET-1 levels in the ECs, we treated the cells with the maximal concentration of curcumin (100 μM) and assayed for ET-1 transcript level by real time RT-PCR. Our results indicate that curcumin potentiated high glucose-induced ET-1 levels in the ECs (Figure 4A).

In order to assess whether curcumin prevents high glucose-induced oxidative stress, we employed the early molecular marker of oxidative damage, HO-1 and HO-2. Our results show that glucose-induced HO-1 and HO-2 expression is normalized by pre-treatment with curcumin (Figure 4B).

## Discussion

In the present study, we showed that diabetes causes an increase in eNOS and iNOS mRNA levels in the cardiac tissues, in association with increased oxidative protein and DNA damage. Treatment of diabetic animals with curcumin prevents diabetes-induced eNOS/iNOS upregulation and oxidative stress. We also report, for the first time, that curcumin potentiates diabetes-induced ET-1 mRNA levels in the heart. Furthermore, the mechanism of NOS alteration by curcumin involves activation of NF-κB and AP-1.

Over the past several decades, researchers have provided substantial evidence that oxidative stress is augmented in diabetic complications, including cardiomyopathy [35,36]. Several pathways have been shown to augment oxidative stress during long-standing diabetes [8,9]. The mechanisms of increased oxidative stress in diabetes are multifactorial [6-9]. One mechanism is by increasing glucose flux through the aldose reductase pathway, resulting in alterations of the cofactors required for several important antioxidant enzymes. Another mechanisms is an increased flux through the mitochondrial electron transport chain; thus, leading to the production of a superoxide anions. Activation of protein kinase C has also been documented as increasing oxidative stress since such activation leads to altered NADPH oxidase function. Other pathways include the generation of advanced glycated end (AGE) products and glucose auto-oxidation. The NO pathway has also been implemented in oxidative stress during diabetic complications [8,9,37]. We have previously shown that the levels of NO in long-standing diabetes are not changed, however, NOS mRNA expression is upregulated [18]. This suggests that early increased NO could be sequestered by scavenger molecules such as superoxide anions and subsequently damage proteins/DNA and increase oxidative stress. Our results of the present study do support this notion. We have shown that diabetes leads to increased eNOS and iNOS expression in association with augmented peroxynitrite/8OHdG levels.

Curcumin, a potent antioxidant and anti-inflammatory compound, has been shown to mediate many biological affects such as downregulation of NOS [38-40]. A recent study showed that treating lipopolysaccharide-activated murine macrophages with curcumin reduced the levels of iNOS mRNA and protein [39]. The researchers provided support that the reduction in iNOS was attributed to inhibition of redox-active transcription factors required for iNOS induction. Curcumin may also directly lead to reduced NO oxidation by sequestering the reaction intermediate, nitrogen dioxide [41]. Our results confirm these findings in the heart and show that curcumin reduces NO scavenging as assessed by lower nitrotyrosine staining levels. We have also shown that both eNOS and iNOS mRNA levels are reduced in curcumin-treated diabetic cardiac tissues. Moreover, we have provided first evidence of a similar effect of curcumin in cultured ECs. These changes were associated with reduced activation of NF-κB and AP-1.

One other interesting finding of the study is the effect of curcumin on ET-1 expression in the heart as well as cultured ECs. ET-1 was found to be further increased in curcumin-treated diabetic animals and ECs exposed to high glucose levels. It is possible that curcumin may have a differential effect on the vasoactive factors. Our results show that curcumin decreases eNOS/iNOS levels in the heart and cultured ECs but increases ET-1 levels. The mechanism of such action and potential significance remains to be determined. The differential effects of curcumin on eNOS/iNOS and ET-1 may be due to the activation of different transcriptional regulators. Another possible explanation may be that the downregulation of eNOS/iNOS could have a secondary effect on ET-1. It is also plausible that such an alteration may represent the importance of tissue microenvironment and bystander cells. In support of such a notion, our studies indicate that curcumin decreases ET-1 levels in the kidneys and the retina (data not shown). Further studies are necessary to elucidate whether such differences represent the different molecular aberrations in the target organs of chronic diabetic complications.

## Conclusion

Our results support the findings that curcumin prevents diabetes-induced oxidative protein and DNA damage, in association with decreasing NOS levels. Our findings also suggest that the use of curcumin in novel therapeutic modalities should be approached with caution, as curcumin may augment vasoactive peptide ET-1 and lead to vascular dysfunction in diabetes.

## Competing interests

The author(s) declare that they have no competing interests.

## Authors' contributions

HF conducted the majority of the *in vitro *and *in vivo *experiments, including helping maintain the diabetic animals. HF also assisted in drafting the manuscript and preparing it for publication.

ZAK maintained the diabetic animals and helped to interpret the *in vivo *and *in vitro *data. ZAK also helped draft the manuscript and revised it critically for intellectual content.

SC helped with conducting the molecular techniques and assisted in interpreting the results.

SC* assisted in the experimental design, revised the manuscript critically for intellectual content, and gave the final approval for the manuscript to be submitted for publication. In addition, all the experiments were conducted under his grant approval.
